# Viable bacterial communities in freshly pumped human milk and their changes during cold storage conditions

**DOI:** 10.1186/s13006-025-00738-0

**Published:** 2025-05-29

**Authors:** Eliska Pivrncova, Jan Bohm, Vojtech Barton, Jana Klanova, Petra Borilova Linhartova

**Affiliations:** RECETOX, Faculty of Science, Masaryk University, Kotlarska 2, Brno, Czech Republic

**Keywords:** Human milk, Microbiome, Viable bacteria, Storage, Propidium monoazide, Next-generation sequencing, 16S rRNA, Milk expression, Pumped milk

## Abstract

**Background:**

Human milk harbors diverse bacterial communities that contribute to infant health. Although pumping and storing milk is a common practice, the viable bacterial composition of pumped milk and the impact of storage practice on these bacteria remains under-explored. This metagenomic observational study aimed to characterize viable bacterial communities in freshly pumped human milk and its changes under different storage conditions.

**Methods:**

In 2023, twelve lactating mothers from the CELSPAC: TNG cohort (Czech Republic) provided freshly pumped milk samples. These samples were stored under various conditions (refrigeration for 24 h, 48 h, or freezing for six weeks) and treated with propidium monoazide (PMA) to selectively identify viable cells. The DNA extracted from individual samples was subsequently analyzed using 16S rRNA amplicon sequencing on the Illumina platform.

**Results:**

The genera *Streptococcus, Staphylococcus, Diaphorobacter, Cutibacterium,* and *Corynebacterium* were the most common viable bacteria in fresh human milk. The median sequencing depth and Shannon index of fresh human milk samples treated with PMA (+ PMA) were significantly lower than in untreated (-PMA) samples (*p* < 0.05 for all), which was true also for each time point. Also, significant changes in these parameters were observed between fresh human milk samples and their paired frozen samples (*p* < 0.05), while no differences were found between fresh human milk samples and those refrigerated for up to 48 h (*p* > 0.05). Of specific genera, only + PMA frozen human milk samples showed a significant decrease in the central log-ratio transformed relative abundances of the genera *Diaphorobacter* and *Cutibacterium* (*p* < 0.05) in comparison to + PMA fresh human milk samples.

**Conclusions:**

The study demonstrated that the bacterial profiles significantly differed between human milk samples treated with PMA, which represent only viable bacteria, and those untreated. While storage at 4 °C for up to 48 h did not significantly alter the overall diversity and composition of viable bacteria in human milk, freezing notably affected both the viability and relative abundances of some bacterial genera.

**Supplementary Information:**

The online version contains supplementary material available at 10.1186/s13006-025-00738-0.

## Background

Understanding the composition of the viable bacterial community in human milk is essential as it underpins numerous health benefits for both mother and infant [1–4]. Therefore, the diversity and composition of bacteria present in human milk is a field of growing interest in the scientific community. Multiple methods can be employed to characterize human milk bacteriota; while some require culture of the samples prior to their analysis, others do not [5]. Culture-independent methods are more comprehensive and can detect a broader range of bacterial genera in human milk compared to culture-dependent methods. However, it is important to note that metagenomic analyses do not differentiate between DNA originating from living or dead bacterial cells [6, 7]. This means that the presence of cell-free DNA and non-viable bacteria can potentially impact the data interpretation. Given the insights provided by metagenomic studies and the potential of viable bacteria in human milk to colonize the oral cavity and gastrointestinal tract of infants, knowing how common practices such as milk pumping and storage might affect these bacterial communities is of great importance. One of the potential methods of detecting viable bacterial cells lies in the incorporation of DNA-intercalating dye propidium monoazide (PMA). PMA works by binding to DNA and preventing PCR amplification of DNA from dead or damaged bacterial cells, thus allowing only DNA from viable cells to be amplified and detected [8]. This method has been used previously in various sample materials [9–11] including human milk [12].

The design of the presented study corresponds to the real-life handling of human milk. While several studies have investigated the impact of pumping and storage on the human milk microbiome [13, 14], there is still a need for further exploration of how these practices specifically influence the abundance, diversity, and viability of milk bacterial communities [15, 16]. Thus, it is very important to investigate the potential benefits and drawbacks of these practices. The primary objective of our study was to characterize the profile of pumped fresh human milk bacteriome, especially from viable bacterial cells, and to characterize the human milk bacteriome under selected storage conditions, namely refrigeration for 24 and 48 h, and freezing for six weeks.

## Methods

**Fig. 1:**
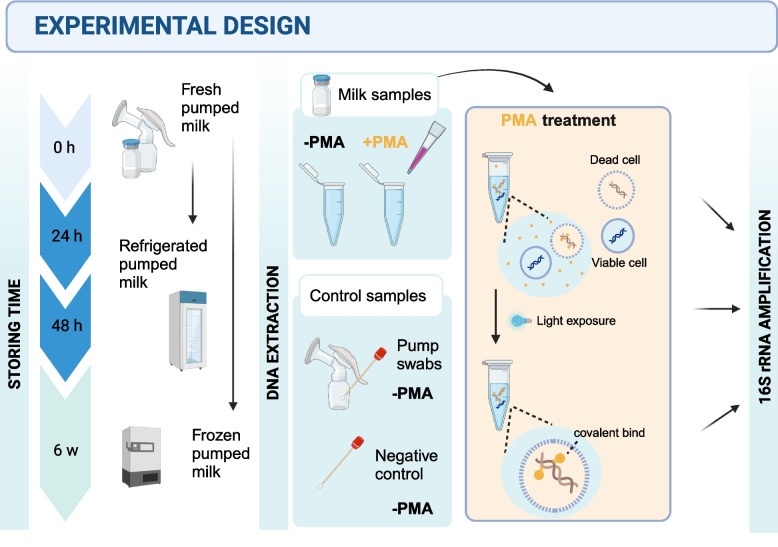
Methodological framework for assessing viable bacterial content in fresh and stored human milk. The framework overview of the treatment with propidium monoazide (PMA) to distinguish between viable and non-viable bacteria, and subsequent storage at different temperatures and durations. -PMA, samples untreated with propidium monoazide; + PMA, samples treated with propidium monoazide

This metagenomic observational study was performed in 2023 (Czech Republic, Brno). To investigate the viable profile of bacterial communities in human milk, we approached lactating mothers willing to provide samples of their freshly pumped milk. Fresh samples, samples refrigerated at 4 °C for 24 h or 48 h, and those frozen at −20 °C for six weeks were analyzed (with and without PMA treatment to distinguish between viable and non-viable cells) in line with our experimental design (Fig. 1).

### Study population

Human milk samples were obtained from a subset of twelve participants in the Central European Longitudinal Studies of Parents and Children: The Next Generation (CELSPAC: TNG). In line with the Helsinki declaration, all women involved in this study were willing to participate and gave informed consent. Only healthy mothers giving birth at ≥ 36 weeks of their pregnancy were included in the study. General exclusion criteria were: personal history of systemic diseases (diabetes mellitus, obesity, asthma cardiovascular diseases, oncological diseases, immunodeficiency), serious peri- or post-labor complications, C-section delivery, cessation of exclusive breastfeeding prior to sample collection, antibiotic treatment and/or mastitis within one month prior to the sample collection, and breastfeeding difficulties that would prevent sample collection. Data related to pregnancy, birth, and mother’s health characteristics were collected through questionnaires.

### Sample collection

Human milk samples were collected using manual breast pumps in a uniform manner by a single lactation consultant at mothers’ households within the period of second to fourth month after the delivery. Manual breast pumps were sterilized at the laboratory, yet control swabs from pump equipment (pump swabs, *n* = 24) for detection of contaminants were taken prior to each use. The instructions for sample collection were as follows: 1) do not breastfeed or express milk for at least 2 h before the sample collection; 2) express at least 20 ml of human milk using the manual breast pump and sterile pump kit; 3) express milk from both breasts, ideally 10 ml from each. Fresh human milk samples (time point, TP 0 h) were immediately transported to the laboratory for further processing (< 30 min in icebox). Each sample was aliquoted (1 ml) and either processed immediately, stored at 4 °C for 24 h (TP 24 h), for 48 h (TP 48 h), or stored at −20 °C for six weeks (TP 6 w). At each time point, two aliquots of human milk samples were analyzed for a DNA profile of viable bacteria only (+ PMA) as well as of all present bacteria (-PMA). Note that these two aliquots from each time point do not represent exact duplicates but rather paired samples for distinguishing between viable and non-viable bacteria.

### PMA treatment and DNA extraction

Firstly, human milk samples, pump swabs, negative controls (sterile DNA-free water, *n* = 22), and ZymoBIOMICS™ Spike-in Control I (High Microbial Load, ZymoResearch, CA), so-called MOCK community, were centrifuged at 10,000 × *g* for 10 min at 4 °C. The supernatant with fat was discarded and the pellet was resuspended in 1 ml of sterile DNA-free water (Qiagen, Germany). 0.5 µl of PMAxx™ (20 mM, Biotium, USA) was added to each PMA-treated sample to a final concentration of 10 µM [7]. PMA concentration was adjusted to 10 μM based on our 16S rRNA amplicon sequencing results from experiments on live and dead cultures. Samples were vortexed for 20 s and incubated in the dark at room temperature for 15 min with vortexing every 5 min [12]. Samples were then exposed to light using a PMA-Lite LED Photolysis Device (Biotium, USA) for 15 min, with vortexing every 5 min. The non-PMA-treated samples were kept at room temperature for the whole duration of the PMA treatment. Afterward, all samples were centrifuged at 5,000 × *g* for 10 min, the supernatant was discarded. The pellet was suspended and DNA extracted using the QIAmp PowerFecal DNA kit (QIAGEN, Germany) on the QIAcube instrument (QIAGEN, Germany) according to the manufacturer’s instructions with addition of 1 µl of Rnase (New England BioLabs, USA). Extracted DNA was stored at −20 °C.

### The 16S rRNA amplicon sequencing

The 16S rRNA library was prepared according to the Illumina 16S metagenomic sequencing library preparation protocol with the PCR reagents decontaminated with 8-methoxypsoralen (8-MOP; 0.16 mM, Sigma-Aldrich, USA) [17]. Each PCR reaction mix was spiked with 1 µl of extracted and diluted MOCK community to ensure optimal sequencing output in low-abundance samples. The total volume of 31 µl consisted of 15 µl of Q5® High Fidelity Master Mix (New England Biolabs, USA), 1.5 µM of each primer (10 uM), 2 µl of 8-MOP 10 × diluted, 1 µl of MOCK community, 5 µl of extracted DNA, and 5 µl of sterile DNA-free water. The V4 region (~ 290 bp) was amplified using F515 (5’-GTGCCAGCMGCCGCGGTAA-3’) and R806 (5’-GGACTACHVGGGTWTCTAAT-3’) primers [18]. The initial denaturation (15 min at 95 °C) was followed by 30 cycles consisting of denaturation at 94 °C for 35 s, primer annealing at 55 °C for 35 s, and extension at 72 °C for 45 s. The final extension at 72 °C lasted 10 min. Each PCR batch consisted of DNA extraction controls (sterile DNA-free water used in extraction), PCR negative controls, pump swabs, human milk samples, and positive controls (a mixture of stool samples with high load of bacterial DNA). PCR products were assessed on 1.5% agarose gel and cleaned with SPRIselect beads (Beckman Coulter Genomics, USA) according to the manufacturer’s instructions. Quant-iT (Thermo Fisher Scientific, USA) and microplate reader Synergy Mx (BioTek, USA) were used to assess the concentration of cleaned PCR products to pool those with different inner tags equimolarly. Pools were indexed with Nextera XT indexes (Illumina, USA), quantified fluorochemically, and pooled equimolarly. The prepared library was assessed with a 2100 Bioanalyzer Instrument (Agilent Technologies, USA) shortly before sequencing. The library was diluted to a final concentration of 10 pM, and 20% of PhiX DNA (Illumina, USA) was added. Sequencing was performed with the Miseq reagent kit V3 using a MiSeq 2000 instrument according to the manufacturer’s instructions (Illumina, USA). Measures to prevent batch effect were employed throughout the entire analysis.

### Bioinformatic analysis

Data were processed using nf-core/ampliseq version 2.7.1 [19] of the nf-core collection of workflows, utilizing reproducible software environments from the Bioconda [20] and Biocontainers projects [21]. Data quality was evaluated with FastQC [22] and summarized with MultiQC [23]. Sequences were processed sample-wise (independent) with DADA2 [24] to eliminate PhiX contamination, trim reads (before median quality drops below 25 and at least 85% of reads are retained; reads shorter than 289 bp for forward reads and 222 bp for reverse reads were removed), discard reads with > 3 expected errors, correct errors, merge read pairs, and remove PCR chimeras; ultimately, 10,446 amplicon sequencing variants (ASVs) were obtained across all samples. Taxonomic classification was performed by DADA2 and the database ‘Silva 138.1 prokaryotic SSU’ [25]. ASV sequences, abundances, and DADA2 taxonomic assignments were loaded into QIIME2 [26]. ASVs containing any mitochondria or chloroplasts were removed. Within QIIME2, the final bacterial community data were visualized in a barplot (not presented). As a quality control step, rarefaction curves were produced to verify sufficient sequencing depth (see Additional files, Supplementary Figure S1).

### Statistical analysis

Biostatistical analyses were carried out using the R programming language, version 4.1.2, released on November 1, 2021 [27]. A significance threshold of 0.05 was applied to all statistical tests, with Holm’s method used to adjust *p*-values for multiple comparisons within each analysis. Taxonomical evaluations were restricted to data classified at the genus level.

In the first step, MOCK reads were removed from the sequencing data. Subsequently, Shannon diversity index (reflecting evenness) was computed at the amplicon sequence variant (ASV) level using the vegan package [28] on data rarefied to 10,000 reads. This rarefaction step was implemented to reduce biases associated with varying sequencing depths. Group differences in sequencing depth and Shannon index were assessed using the Wilcoxon test (applying the paired version when appropriate).

A Bray–Curtis dissimilarity matrix was computed to compare the bacteriome profiles across all samples. Then, for each milk or pump swab sample, a one-sided Wilcoxon test was performed, comparing the distribution of Bray–Curtis dissimilarity between that sample and other (non-control) samples to the distribution of dissimilarity values between that sample and the negative controls. After adjusting the *p*-values for multiple comparisons, samples for which the null hypothesis was rejected were classified as having bacteriome profiles similar to those of the negative controls.

To compare presence/absence of given genera between human milk samples and control samples, a proportion test was used. In this case, due to low statistical power, presented results are not adjusted for multiple hypothesis testing. For bacteriome composition, three distinct generalized linear models were constructed using ALDEx2 (ANOVA-Like Differential Expression) tool for high throughput sequencing data [29] to account for the compositional nature of the data through centered log-ratio (CLR) transformation. These models were defined as follows:

- Model 1: Comparison between human milk samples and control samples.
- Model 2: Comparison between PMA-treated and non-PMA-treated human milk samples across all TPs.
- Model 3: Comparison among different storing conditions in + PMA human milk.

For visualization, mean CLR-transformed abundance values were obtained using the aldex.clr function with 256 Monte Carlo replicates. Beta diversity was evaluated using the Principal Coordinates Analysis (PCoA) using the robust Aitchison distance (a method based on a centered log-ratio, CLR, transformation well-suited for the analysis of compositional data) computed in the ape package [30]. Graphical representations of these analyses were generated using ggplot2 [31], ggsignif [32], and ggstatsplot [33]. Additionally, a heatmap displaying CLR-transformed values for the 25 most abundant bacterial genera (by total relative abundance) was created with the ComplexHeatmap package [34].

## Results

Data related to pregnancy, birth, and mother’s health characteristics are shown in Table 1. In total, 142 samples (96 human milk samples, 24 pump swabs, 22 negative controls) were processed and analyzed. Descriptive data on the sequencing depths, Shannon indices, and CLR-transformed relative abundances of genera mentioned in this text are presented in the Additional files, Supplementary Figure S2.

**Table 1:** Data related to pregnancy, birth, and mother’s health characteristics (*n* = 12)

Mother’s characteristics	n
Age at delivery in years (mean ± SD)	34.57 (± 4.72)
Week of delivery (mean ± SD)	40.28 (± 0.75)
Intrapartum antibiotic prophylaxis (GBS-positive)	1
Postpartum antibiotic use^a^	3
History of postpartum mastitis^a^	1
Probiotic use postpartum	5

*SD* standard deviation, *GBS* Group B *Streptococcus* positivity, *n* number

^a^ More than one month prior to sample collection

### Comparison of human milk samples and control samples

**Fig. 2:**
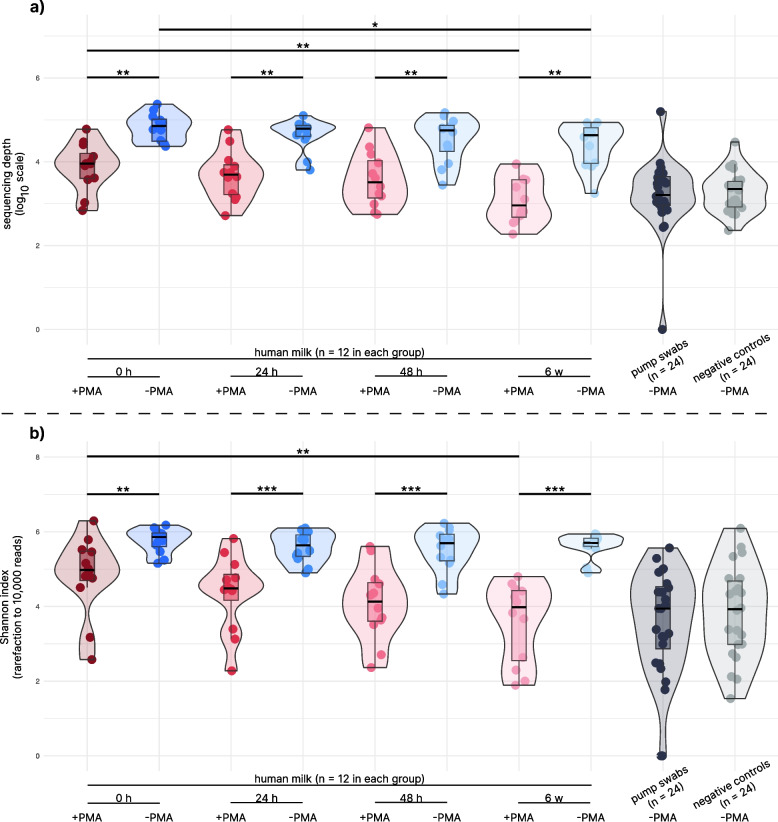
Violin boxplots of sequencing depth (**a**) and Shannon index (**b**) in human milk samples under different storage conditions. This figure shows the sequencing depth and Shannon diversity index of human milk samples stored under three conditions: at 4 °C for 24 h, at 4 °C for 48 h, and at −20 °C for six weeks. Both PMA-treated samples (+ PMA, representing only viable genera) and untreated samples (-PMA, representing both viable and non-viable bacterial genera) are included. The horizontal line inside each box represents the median, while the box indicates the interquartile range. Sequencing depth values are log₁₀-transformed, and the Shannon index was calculated on data rarefied to 10,000 reads. Group comparisons were performed using the Wilcoxon test (paired when applicable). Although comparisons with negative controls and pump swabs yielded significant differences, those comparisons were omitted from the final plot for clarity. *P*-values were adjusted using Holm’s method, with significance indicated as follows: ٭ *p* < 0.05, ٭٭ *p* < 0.01, and ٭٭٭ *p* < 0.001

The sequencing depth and Shannon index of both pump swabs and negative controls were significantly lower than those observed in fresh human milk samples (both + PMA and -PMA; Wilcoxon paired test, *p*_ADJ_ < 0.01, see Fig. 2), indicating the fact that the bacterial load in control samples and the contamination were very low. No differences in the CLR-transformed relative abundances of any of the genera were observed between pump swabs and negative controls, indicating a strong similarity between these control samples. Relative abundances of the genera *Streptococcus* and *Gemella* in -PMA fresh human milk samples were significantly higher than in both pump swabs and negative controls (ALDEx2, *p*_ADJ_ < 0.05). In terms of the presence/absence of given bacterial genera, the human milk samples were also enriched in *Rothia* or *Anaerococcus* and lack *Bacteroides* compared to both groups of control samples (proportion tests, *p* < 0.05). In addition, increased presence of the genera *Bacillus, Acinetobacter, Cutibacterium, Veillonella*, and *Staphylococcus* was observed in human milk samples compared to negative controls (proportion tests, *p* < 0.05).

**Fig. 3:**
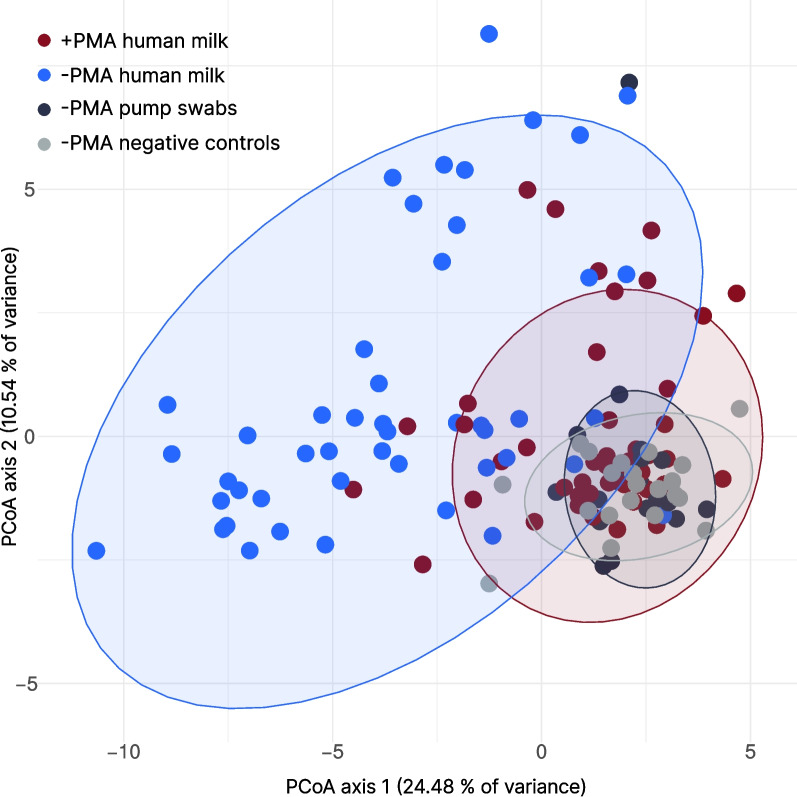
Principal coordinates analysis (PCoA) of human milk samples with and without PMA treatment. This figure illustrates PCoA based on robust Aitchison distance at the genus level on human milk samples without any additional treatment (-PMA, *n* = 48) and human milk samples treated with propidium monoazide (+ PMA, *n* = 48) in comparison to PMA control samples (pump swabs, *n* = 24; negative controls – DNA-free water, *n* = 22)

The 25 most abundant genera, represented as CLR-transformed relative abundances, are depicted in the heatmap (Additional files, Supplementary Figure S3), which clearly illustrates the similarity between pump swabs and negative controls. This similarity is also apparent in the PCoA plot based on robust Aitchison distances (Fig. 3).

Of all 96 human milk samples, the bacteriome profile of only five of them (all from the + PMA group) matched the profile of our negative controls. Unexpectedly, only seven of the pump swabs were highly similar in bacteriome profile to negative controls. Table 2 shows the list of bacterial genera present in more than half of the samples of + PMA fresh human milk, -PMA fresh human milk, pump swabs and negative controls, and their mean and median relative abundances. Of those, genera *Bacillus* and *Veillonella* were often present in both human milk samples and pump swabs but not so often in negative controls.

**Table 2:** Bacterial genera detected in more than 50% of samples for each sample type and their relative abundances

;-PMA fresh human milk (*n* = 12)	; + PMA fresh human milk (*n* = 12)	;-PMA pump swabs (*n* = 24)	;-PMA negative controls (*n* = 22)
*Streptococcus* (12; 47.5%; 56.9%)	*Streptococcus* (12; 21.4%; 14.6%)	*Streptococcus* (18; 7.7%; 4.7%)	*Streptococcus* (19; 13.1%; 15.3%)
*Staphylococcus* (12; 11.3%; 10.8%)	*Staphylococcus* (12; 16.4%; 12.0%)	*Staphylococcus* (18; 7.3%; 4.9%)	*Staphylococcus* (19; 10.1%; 7.1%)
*Gemella* (11; 1.8%; 1.0%)			
*Corynebacterium* (11; 0.6%; 0.4%)	*Corynebacterium* (10; 2.1%; 0.9%)	*Corynebacterium* (15; 4.1%; 0.9%)	*Corynebacterium* (14; 3.4%; 1.5%)
*Cutibacterium* (11; 0.7%; 0.4%)	*Cutibacterium* (11; 2.0%; 1.3%)		
*Veillonella* (10; 2.3%; 0.5%)	*Veillonella* (8; 4.7%; 1.0%)	*Veillonella* (13; 2.8%; 1.4%)	
*Rothia* (10; 3.8%; 3.9%)	*Rothia* (8; 1.2%; 0.4%)		
*Haemophilus* (9; 0.3%; < 0.1%)			
*Diaphorobacter* (9; 5.4%; 0.1%)	*Diaphorobacter* (12; 3.4%; 1.9%)		
*Actinomyces* (8; 1.9%; 0.5%)			
*Acinetobacter* (8; 0.1%; < 0.1%)			
*Micrococcus* (8; 0.3%; 0.1%)		*Micrococcus* (14; 12.7%; 2.4%)	*Micrococcus* (13; 5.8%; 2.5%)
*Bacillus* (7; 12.0%; 0.1%)	*Bacillus* (7; 14.4%; 0.2%)	*Bacillus* (14; 7.5%; 2.4%)	
*Anaerococcus* (7; 0.1%; < 0.1%)			
*Kocuria* (7; 0.4%; 0.1%)	*Kocuria* (8; 1.6%; 0.5%)	*Kocuria* (14; 4.4%; 1.6%)	*Kocuria* (13; 9.5%; 3.2%)
*Enhydrobacter* (7; 0.1%; < 0.1%)	*Enhydrobacter* (7; 0.2%; 0.1%)		
	*Sphingomonas* (7; 0.5%; 0.2%)		

A genus was considered present in a sample if its relative abundance was greater than 0. Numbers in parentheses indicate the number of samples within each group in which the genus was detected and the mean and median relative abundances, respectively

-PMA, samples untreated with propidium monoazide; + PMA, samples treated with propidium monoazide

### Characterization of viable bacteriome profiles in human milk

All fresh human milk samples contained bacteria of the genus *Streptococcus* and *Staphylococcus*. All bacterial genera present in more than half of those samples are listed in Table 2, with *Streptococcus, Staphylococcus, Diaphorobacter, Cutibacterium,* and *Corynebacterium* being the most common and abundant in fresh human milk.

Significant differences between + PMA (viable) and -PMA (non-viable) human milk samples from were detected both in sequencing depths and Shannon indices; -PMA samples had higher sequencing depths as well as Shannon indices than + PMA samples (Wilcoxon paired tests, *p*_ADJ_ < 0.01 for all, see Fig. 2).

PCoA (Fig. 3) showed that + PMA human milk bacteriome composition was more similar to the bacteriome composition of pump swabs and/or negative controls. + PMA samples have reduced variability compared to the -PMA samples, pump swabs and/or negative controls.

**Fig. 4:**
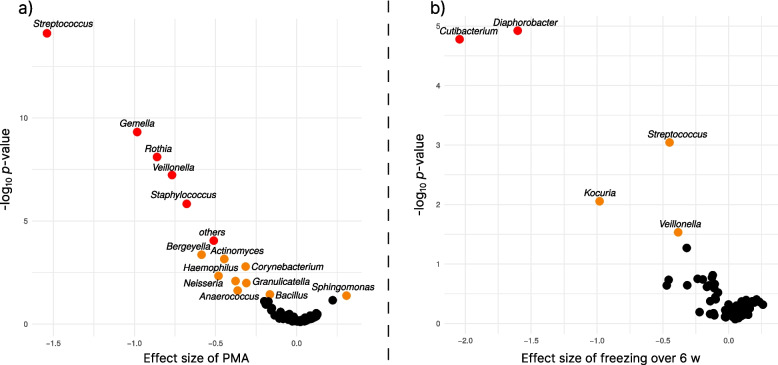
Volcano plots of bacterial composition changes. Volcano plots show the effect sizes and ***p***-values from generalized linear models using the ALDEx2 framework that explore the influence of propidium monoazide (PMA) treatment (**a**) and freezing for six weeks (**b**) on the bacterial composition of milk. Only significantly associated genera are described; orange markers indicate significant *p*-values before Holm’s adjustment, and red markers denote associations that remain significant after *p*-value adjustment

The bacteriome profiles varied individually among samples, see Additional files, Supplementary Figure S3. Notably, relative abundances of bacterial genera *Streptococcus, Gemella, Staphylococcus, Veillonella*, and *Rothia* (ALDEx2, *p*_ADJ_ < 0.001) were significantly reduced in + PMA human milk samples compared to -PMA human milk samples, see the volcano plot in Fig. 4a. The statistically significant decrease in the proportion of unassigned reads marked as “others” (ALDEx2, *p*_ADJ_ = 0.019) in + PMA human milk samples in comparison to -PMA human milk samples is another interesting outcome, suggesting that PMA treatment improves the assignment accuracy by reducing interference from extracellular DNA.

### Effect of storage conditions on human milk bacteriome

In comparison to paired fresh human milk samples, the median sequencing depths were significantly lower in human milk samples frozen for six weeks (Wilcoxon paired test, *p*_ADJ_ < 0.01) for both PMA-treated and untreated samples, see Fig. 2a. Additionally, the Shannon index decreased in + PMA human milk samples frozen for six weeks (Wilcoxon paired test, *p*_ADJ_ < 0.01) compared to the fresh ones, see Fig. 2b. No significant differences in these parameters were observed between fresh human milk samples and paired refrigerated samples stored for 24/48 h (*p* > 0.05; this was true for both + PMA and -PMA samples).

The comparative analysis of relative abundances of bacterial genera focused on + PMA human milk samples. Over six weeks at −20 °C, the relative abundance of the genera *Diaphorobacter* and *Cutibacterium* significantly decreased (ADLEx2, *p*_ADJ_ = 0.003 and *p*_ADJ_ = 0.007) in comparison to paired fresh human milk samples, see Fig. 4b for the ALDEx2 result.

## Discussion

Our results revealed that human milk samples treated with PMA exhibited a decreased number of detected reads compared to untreated samples. Our study builds upon previous findings, especially those by Stinson et al. [12], by specifically characterizing the viable bacterial community in fresh pumped human milk. In the presented study, we have confirmed their results and supplemented them with the investigation of the bacteriome changes in human milk under real-life storage conditions. This approach provides novel insights into the impact of common storage practices on human milk quality.

### Viable bacteria in fresh human milk

In our study, some interpersonal variability in human milk bacteriome was detected. Nevertheless, most of the samples resembled human milk microbiota profiles identified by other studies using both culture-dependent and culture-independent methods [35–41]. In line with our findings, previous studies have identified *Streptococcus*, *Staphylococcus*, and *Cutibacterium* (formerly *Propionibacterium*), as the predominant genera in human milk, noting their universal presence in milk microbiota regardless of the geographical location or the analytical techniques applied [12, 13, 37, 38, 41, 42].

Bacterial communities as well as bacterial DNA in human milk are often transferred from one habitat to another within an individual and mother-infant pair. First of all, it has been proposed that immune cells can translocate bacteria from the maternal gut to the mammary glands [43]. It is also known that genera *Staphylococcus, Streptococcus, Corynebacterium,* *Cutibacterium* and *Pseudomonas* are typical of maternal and/or infant skin [44], and all these were also found in our human milk samples. Moreover, the retrograde flow of milk from the infant's mouth to the mammary gland was documented [45], which may also contribute to the dominance of oral *Streptococcus* species. Furthermore, other bacterial genera common for the oral cavity, specifically *Rothia, Gemella*, *Veillonella*, *Actinomyces*, and *Granulicatella*, were found in human milk samples in our study. This suggests a transition of bacterial cells and DNA from the infant’s oral cavity and/or skin on mother’s breast. In the case of the genus *Streptococcus*, we observed a significant difference in the relative abundances of viable and non-viable bacteria in human milk. We can hypothesize that this relatively high proportion of non-viable *Streptococcus* cells might be caused by the more adverse conditions for their survival on the breast surface compared to the infant’s oral environment; a definite explanation of this, however, requires further research.

Contamination of human milk samples (which are characterized by low biomass and, therefore, particularly prone to be influenced by contamination) during collection, together with reagent contamination, are among the major pitfalls of this type of study [46, 47]. To prevent this issue, we used carefully designed control samples to identify the sources of contamination. This design helped us determine that contamination came from both reagents and equipment used for expressing milk and collecting samples, even when the equipment was supposed to be sterile.

The genus *Bacillus* was present more often in human milk samples and in the pump swabs than in negative controls. *Bacillus* spp. is a common contaminant of human milk, there is even an operating protocol to avoid contamination of donor human milk with *Bacillus cereus* (a pathogen causing emetic and diarrheal diseases) published by the official journal of the International Lactation Consultant Association [48]. Urbaniak et al. also noted the presence of *Bacillus* in human mammary tissue (together with *Acinetobacter, Pseudomonas*, *Staphylococcus, Prevotella,* or *Cutibacterium*) [49]. In addition, Jiménez et al. reported higher concentrations of some contaminant bacteria (particularly *Pseudomonas* spp.) in their samples [50]. These studies demonstrate that we should distinguish between the profiles of pumped human milk and manually expressed milk [42].

Based on negative controls, Leech et al. identified bacteria *Porphyromonas gingivalis, Enterococcus faecalis, Streptococcus mutans, Escherichia coli,* and *Acinetobacter baylyi* as contaminants in their study. Importantly, these five species made up on average 83% of the total bacterial content detected in each of the human milk samples in their study [51]. Reyes et al. also reported that human milk pumped using the equipment brought by the patients from their homes contained significantly higher relative abundances of *Acinetobacter* than samples collected using hospital-provided sterile breast pumps [52]. The genera *Pseudomonas* and *Acinetobacter* were also detected in our samples (both pump swabs and human milk samples); their detection was, however, relatively rare (in less than 50% of samples). Identifying potential pathogenic bacteria may have important clinical implications. For instance, *Acinetobacter* species have been linked to illness in preterm infants [53]. Additionally, Leech et al. also challenged the evidence for the existence of human milk microbiota based on their results, in which the bacterial biomass in human milk samples was very low, comparable with negative (PCR no-template and extraction) controls [51]. Some previously published studies even found no bacteria in human milk samples at all [54, 55]. In our study, however, the bacteriome profile similar to negative controls was detected only in one fresh human milk sample.

It is crucial to clarify the perspective from which we intend to define the human milk microbiome, i.e., whether we consider it as the pure milk microbiome present in the milk ducts, or as the comprehensive profile of the milk ingested by the infant (which includes the microbiome from the mother's skin and the infant's oral cavity). In the context of expressed human milk, which is commonly used in practice, we consider it important to take bacteria from the breast pump into account as a part of the microbiome the infant is exposed to.

### Human milk storage

The use of breast pumps and subsequent storage of pumped human milk is a widespread practice. Moreover, it is a common practice for women to store their milk in the refrigerator and then freeze it if the milk is not used after a few days. Therefore, our study specifically explored how storage temperature and duration influence the bacterial abundance and diversity. Even though the recommendation for pumped milk storage varies from 4–8 days in a refrigerator and 3–12 months in a freezer [56–58], we choose to investigate the effect of storing pumped human milk for 24 h and 48 h in a refrigerator and six weeks in the freezer. These intervals are mostly favored by mothers in European countries [59].

Our results demonstrate significant differences between + PMA and -PMA samples across all examined intervals. This is consistent with the study by Stinson et al. who also reported a significant reduction in the total quantity of DNA from viable cells during cold storage [14]. Moreover, our results showed that freezing human milk at −20 °C for six weeks significantly impacted the sequencing depth, Shannon index, and bacteriome composition of viable bacteria. On the other hand, our results showed no significant changes in these parameters after two days of human milk refrigeration at 4 °C. Still, however, it is important to consider the potential for the growth of psychrotrophic bacteria (i.e. bacteria capable of thriving at temperatures of 7 °C or lower), such as *Bacillus* sp., under these conditions, which might be hazardous for the infant [60]. This highlights the need for careful handling and timely use of refrigerated human milk to minimize potential health risks.

### Strengths and limitations

Our study significantly contributes to the field by offering a comprehensive method to characterize the viable human milk microbiome. This characterization is particularly critical when integrating results with metatranscriptomic and metabolomic data, allowing the holistic assessment of the potential impact of the microbial community in human milk on both maternal and infant health.

The approach to sample collection is one of the particular strengths of this study. The inclusion of a MOCK community in PCR gave us the advantage of the capability to sequence even low-abundance samples. When analyzing low-biomass samples, incorporating a sufficient number of negative controls is essential for the accurate identification of potential contaminants from reagents or pump equipment. In our study, this careful inclusion allowed us to determine if the samples closely mirrored the profile of the negative controls and to assess whether they possessed a unique bacterial community or whether they predominantly comprised of contaminants. This critical step of utilizing negative controls to identify bacterial contaminants in low-biomass samples is overlooked in many existing studies.

However, the study is not without limitations. The sample size, although adequate for preliminary analysis, could be expanded in future studies to achieve greater robustness and statistical power. The use of short 16S rRNA amplicons (∼300 bp) for the analysis of the human milk composition restricted bacterial identification to the genus level. Additionally, while we have effectively captured the bacterial profile of the human milk, distinguishing the origin of these microorganisms—whether intrinsic to the human milk or introduced from the mother’s skin—remains a challenge.

The possible limited efficiency of PMA treatment in human milk samples due to the turbidity of samples might pose another limitation. The high turbidity impacts the optical density and thus may affect the photoactivation of PMA. Nevertheless, the consistent detection of significant differences between + PMA and -PMA samples at each time point suggests that PMA treatment was effective in detecting viable microbial populations.

## Conclusions

In conclusion, the present study analyzed viable bacterial communities in pumped human milk under different storage conditions. The use of PMA enabled the description of the viable bacteria of human milk, revealing that a significant proportion of the detected bacterial DNA in -PMA human milk samples originated from non-viable cells. The most common viable bacteria in freshly pumped human milk included the genera *Streptococcus, Staphylococcus, Diaphorobacter, Cutibacterium,* and *Corynebacterium*. Our findings also reveal that while two-day storage at 4 °C does not significantly alter the overall diversity and composition of viable bacteria in human milk, freezing notably affects the viability and relative abundances of certain bacterial genera, such as *Cutibacterium* and *Diaphorobacter*. While pumping and storing human milk are necessary practices for many families, this study underscores the importance of considering the impact of these practices on the milk's bacterial quality. Future research should continue to explore the health implications of these findings, which could help to optimize milk storage guidelines in a way that preserves the beneficial bacterial communities in human milk.

## Supplementary Information

Additional file 1: Figure S1. Sequencing depth with alpha rarefaction curves in all analyzed samples (*n* = 142). Samples with PMA treatment (+ PMA; only viable genera) as well as without PMA treatment (-PMA; both viable and non-viable bacterial genera) are shown. Figure S2. Descriptive statistics for sequencing depth, Shannon diversity index, and centered log-ratio (CLR)–transformed relative abundances of selected genera, stratified by sample matrix, time point, and PMA treatment group. All values are rounded to two decimals. Figure S3. Triangle heatmap of viable and non-viable bacterial genera in human milk samples. This heatmap displays the genus-level microbial composition of fresh human milk samples and milk samples stored under different conditions, i.e., at 4 °C for 24 and 48 h, and at -20 °C for six weeks. The relative abundances have been transformed using a centered log-ratio (CLR) approach. The top annotation provides a paired comparison of the Shannon index (calculated after rarefaction to 10,000 reads) and the sequencing depth (displayed on a log₁₀ scale, note that the blue and red bars overlap). In the split cells, the top-left triangle represents samples without PMA treatment (-PMA), while the bottom-right triangle represents PMA-treated samples (+ PMA).

## Data Availability

The data supporting the findings of this study are available in the European Nucleotide Archive (ENA) under the accession number PRJEB76042.

## Abbreviations

16S rRNA: 16S Ribosomal RNA
ASVs: Amplicon sequencing variants
CELSPAC: TNG – Central European Longitudinal Studies of Parents and Children—The Next Generation
CLR: Centered log-ratio (transformation)
MOCK: ZymoBIOMICS™ Spike-in control I (High microbial load)
PCoA: Principal coordinates analysis
PCR: Polymerase chain reaction
PMA: Propidium monoazide
RECETOX: Research Centre for Toxic Compounds in the Environment
TP: Time point
