# Supplementary material for: Viable bacterial communities in freshly pumped human milk and their changes during cold storage conditions
Source: Int Breastfeed J. 2025 May 29;20:44. doi: 10.1186/s13006-025-00738-0 (PMC12123816; doi:10.1186/s13006-025-00738-0)
Supplement: Supplementary file 1 — Additional file 1: Figure S1. Sequencing depth with alpha rarefaction curves in all analyzed samples (n = 142). Samples with PMA treatment (+ PMA; only viable genera) as well as without PMA treatment (-PMA; both viable and non-viable bacterial genera) are shown. Figure S2. Descriptive statistics for sequencing depth, Shannon diversity index, and centered log-ratio (CLR)–transformed relative abundances of selected genera, stratified by sample matrix, time point, and PMA treatment group. All values are rounded to two decimals. Figure S3. Triangle heatmap of viable and non-viable bacterial genera in human milk samples. This heatmap displays the genus-level microbial composition of fresh human milk samples and milk samples stored under different conditions, i.e., at 4 °C for 24 and 48 h, and at -20 °C for six weeks. The relative abundances have been transformed using a centered log-ratio (CLR) approach. The top annotation provides a paired comparison of the Shannon index (calculated after rarefaction to 10,000 reads) and the sequencing depth (displayed on a log₁₀ scale, note that the blue and red bars overlap). In the split cells, the top-left triangle represents samples without PMA treatment (-PMA), while the bottom-right triangle represents PMA-treated samples (+ PMA). [file 13006_2025_738_MOESM1_ESM.pdf]

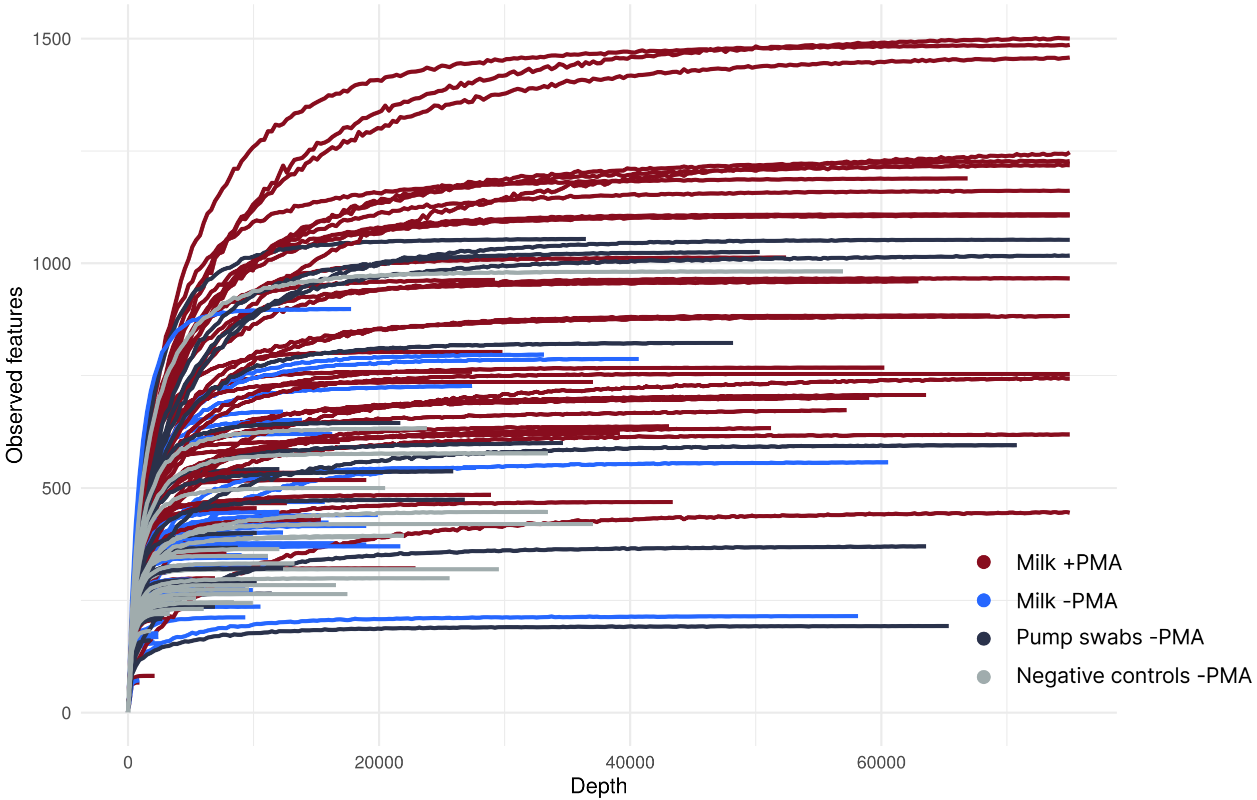

Supplementary Figure S1. Sequencing depth with alpha rarefaction curves in all analyzed samples (n = 142). Samples with PMA treatment (+PMA; only viable genera) as well as without PMA treatment (-PMA; both viable and non-viable bacterial genera) are shown.

<table>
<tr><th></th><th></th><th colspan="8">human milk</th><th></th><th></th><th></th></tr>
<tr><th></th><th></th><th colspan="2">0 h</th><th colspan="2">24 h</th><th colspan="2">48 h</th><th colspan="2">6 weeks</th><th>pump swabs</th><th>negative controls</th><th></th></tr>
<tr><th>total
n = 142</th><th>label</th><th>+PMA
n = 12 (8.5%)</th><th>-PMA
n = 12 (8.5%)</th><th>+PMA
n = 12 (8.5%)</th><th>-PMA
n = 12 (8.5%)</th><th>+PMA
n = 12 (8.5%)</th><th>-PMA
n = 12 (8.5%)</th><th>+PMA
n = 12 (8.5%)</th><th>-PMA
n = 12 (8.5%)</th><th>-PMA
n = 24 (16.9%)</th><th>-PMA
n = 22 (15.5%)</th><th>p-value</th></tr>
<tr><td colspan="13">sequencing depth</td></tr>
<tr><td>[0 | 1440 | 5959.5 | 34602 | 236697]</td><td>five number summary</td><td>[687 | 3923 | 9003.5 | 19490 | 60027]</td><td>[23342 | 29982 | 72746 | 109257.5 | 236697]</td><td>[518 | 1589.5 | 4950.5 | 9283.5 | 58138]</td><td>[6369 | 38159.5 | 61510.5 | 74114.5 | 125347]</td><td>[557 | 1249.5 | 3333 | 12312.5 | 64698]</td><td>[2780 | 15560.5 | 56298 | 80459 | 148187]</td><td>[187 | 448 | 965.5 | 3781.5 | 8890]</td><td>[1753 | 8956 | 43313 | 66746 | 86622]</td><td>[0 | 876 | 1619 | 4751.5 | 157290]</td><td>[229 | 803 | 2242 | 3548 | 29572]</td><td><0.001</td></tr>
<tr><td>25658.81±40063.76</td><td>mean±sd</td><td>14562.92±16904.53</td><td>85814.17±65165.99</td><td>11022.08±16947.94</td><td>56355.5±32357.58</td><td>10766.67±18297.46</td><td>56371.08±47246.3</td><td>2636±3143.96</td><td>41003.75±30897.43</td><td>9055.79±31665.87</td><td>3810.27±6223.63</td><td><0.001</td></tr>
<tr><td colspan="13">Shannon index</td></tr>
<tr><td>[0 | 3.7 | 4.75 | 5.61 | 6.29]</td><td>five number summary</td><td>[2.58 | 4.63 | 4.98 | 5.49 | 6.29]</td><td>[5.16 | 5.56 | 5.85 | 5.97 | 6.18]</td><td>[2.28 | 3.91 | 4.49 | 4.95 | 5.82]</td><td>[4.9 | 5.32 | 5.63 | 5.95 | 6.1]</td><td>[2.36 | 3.58 | 4.13 | 4.67 | 5.61]</td><td>[4.33 | 5.2 | 5.7 | 5.97 | 6.23]</td><td>[1.89 | 2.47 | 3.98 | 4.44 | 4.8]</td><td>[4.9 | 5.59 | 5.7 | 5.83 | 5.94]</td><td>[0 | 2.74 | 3.95 | 4.56 | 5.57]</td><td>[1.53 | 2.98 | 3.93 | 4.75 | 6.09]</td><td><0.001</td></tr>
<tr><td>4.53±1.28</td><td>mean±sd</td><td>4.83±1.05</td><td>5.76±0.33</td><td>4.38±1</td><td>5.59±0.41</td><td>4.08±0.98</td><td>5.51±0.6</td><td>3.58±1.08</td><td>5.63±0.33</td><td>3.62±1.32</td><td>3.84±1.24</td><td><0.001</td></tr>
<tr><td colspan="13">Acinetobacter (CLR transformation)</td></tr>
<tr><td>[-1.27 | -0.56 | -0.2 | 7.16 | 15.4]</td><td>five number summary</td><td>[-0.88 | -0.58 | -0.2 | 6.71 | 9.78]</td><td>[-1.15 | -0.56 | 6.56 | 8.79 | 9.65]</td><td>[-0.91 | -0.54 | -0.32 | 7.68 | 10.29]</td><td>[-0.78 | 3.03 | 7.37 | 8.09 | 11.09]</td><td>[-0.7 | -0.18 | 6.53 | 8.24 | 15.4]</td><td>[-0.9 | -0.6 | -0.17 | 8.03 | 9.41]</td><td>[-0.67 | -0.45 | -0.21 | -0.13 | 0.09]</td><td>[-0.94 | -0.8 | -0.62 | -0.39 | 6.3]</td><td>[-1.13 | -0.41 | -0.21 | 6.44 | 12.14]</td><td>[-1.27 | -0.56 | -0.28 | -0.04 | 11.41]</td><td>0.1</td></tr>
<tr><td>2.61±4.34</td><td>mean±sd</td><td>2.94±4.4</td><td>4.88±4.41</td><td>2.59±4.6</td><td>5.81±4.05</td><td>5.14±5.32</td><td>3.07±4.52</td><td>-0.27±0.23</td><td>-0.04±2.01</td><td>2.5±4.45</td><td>0.97±3.59</td><td><0.001</td></tr>
<tr><td colspan="13">Anaerococcus (CLR transformation)</td></tr>
<tr><td>[-1.37 | -0.58 | -0.3 | 0.21 | 10.54]</td><td>five number summary</td><td>[-0.83 | -0.57 | -0.33 | 7.04 | 10.38]</td><td>[-1.13 | -0.69 | 6.5 | 8.51 | 10.51]</td><td>[-1 | -0.55 | -0.46 | -0.28 | 9.12]</td><td>[-0.92 | -0.77 | -0.53 | 7.09 | 8.27]</td><td>[-0.92 | -0.74 | -0.45 | -0.24 | 8.93]</td><td>[-0.99 | -0.47 | 3.41 | 8.1 | 10.31]</td><td>[-0.51 | -0.19 | -0.13 | -0.03 | 0.21]</td><td>[-1.29 | -0.62 | 2.74 | 8.47 | 10.54]</td><td>[-1 | -0.73 | -0.26 | -0.11 | 10.02]</td><td>[-1.37 | -0.49 | -0.35 | -0.29 | 8.06]</td><td>0.519</td></tr>
<tr><td>1.57±3.74</td><td>mean±sd</td><td>2.92±4.41</td><td>4.42±4.69</td><td>0.77±3.12</td><td>2.1±4.12</td><td>0.26±2.74</td><td>3.88±4.66</td><td>-0.13±0.19</td><td>3.63±4.71</td><td>0.43±2.87</td><td>-0.07±1.85</td><td>0.007</td></tr>
<tr><td colspan="13">Bacillus (CLR transformation)</td></tr>
<tr><td>[-1.42 | -0.44 | 6.93 | 9.36 | 19.53]</td><td>five number summary</td><td>[-0.59 | -0.48 | 6.67 | 9.67 | 16.72]</td><td>[-1.21 | -0.74 | 7.3 | 12.86 | 18.38]</td><td>[-0.58 | -0.39 | 7.92 | 10.9 | 17.07]</td><td>[-0.94 | -0.58 | 7.81 | 11.51 | 17.56]</td><td>[-0.89 | -0.56 | -0.17 | 10.32 | 16.17]</td><td>[-0.8 | -0.21 | 8.35 | 10.22 | 19.15]</td><td>[-0.36 | -0.13 | -0.03 | 8.46 | 15.34]</td><td>[-1.16 | 3.14 | 8.22 | 9.23 | 18.33]</td><td>[-0.71 | -0.09 | 7.88 | 9.38 | 19.53]</td><td>[-1.42 | -0.47 | -0.16 | 7.56 | 11.02]</td><td>0.768</td></tr>
<tr><td>5.39±6.2</td><td>mean±sd</td><td>5.8±6.27</td><td>6.73±7.63</td><td>6.43±6.59</td><td>6.63±7.14</td><td>4.48±6.4</td><td>7.37±6.82</td><td>3.36±5.42</td><td>7.7±6.37</td><td>5.59±5.54</td><td>2.26±4.52</td><td>0.519</td></tr>
<tr><td colspan="13">Bacteroides (CLR transformation)</td></tr>
<tr><td>[-1.35 | -0.76 | -0.44 | -0.11 | 9.33]</td><td>five number summary</td><td>[-0.89 | -0.78 | -0.41 | 2.87 | 8.61]</td><td>[-1.13 | -1.04 | -0.72 | -0.3 | 8.93]</td><td>[-0.98 | -0.67 | -0.63 | -0.5 | -0.26]</td><td>[-1.35 | -1.18 | -0.78 | -0.5 | 0.03]</td><td>[-0.87 | -0.45 | -0.33 | -0.26 | 7.78]</td><td>[-1.22 | -1.1 | -0.77 | -0.43 | -0.06]</td><td>[-0.76 | -0.32 | -0.25 | -0.08 | 0.1]</td><td>[-0.98 | -0.89 | -0.43 | -0.18 | -0.11]</td><td>[-1.09 | -0.6 | -0.3 | 3.52 | 9.33]</td><td>[-1.08 | -0.58 | -0.22 | 0 | 8.97]</td><td>0.007</td></tr>
<tr><td>0.45±2.73</td><td>mean±sd</td><td>1.3±3.48</td><td>0.64±3.32</td><td>-0.6±0.18</td><td>-0.78±0.47</td><td>0.28±2.37</td><td>-0.74±0.39</td><td>-0.24±0.24</td><td>-0.5±0.34</td><td>1.71±3.87</td><td>1.38±3.49</td><td>0.001</td></tr>
<tr><td colspan="13">Cutibacterium (CLR transformation)</td></tr>
<tr><td>[-0.86 | -0.26 | 7.53 | 9.65 | 11.99]</td><td>five number summary</td><td>[-0.32 | 7.59 | 8.37 | 9.29 | 10.61]</td><td>[-0.41 | 8.85 | 10.43 | 10.77 | 11.59]</td><td>[7.21 | 7.72 | 8.23 | 9.39 | 11.41]</td><td>[6.34 | 7.54 | 9.16 | 10.54 | 11.99]</td><td>[-0.78 | 5.82 | 7.15 | 9.45 | 10.73]</td><td>[-0.54 | 7.31 | 9.4 | 10.57 | 11.5]</td><td>[-0.51 | -0.28 | -0.23 | -0.15 | 0.01]</td><td>[-0.86 | -0.8 | -0.62 | -0.27 | -0.06]</td><td>[-0.69 | -0.45 | -0.16 | 9.48 | 11.94]</td><td>[-0.73 | -0.22 | 0 | 9.25 | 10.68]</td><td><0.001</td></tr>
<tr><td>5.39±4.82</td><td>mean±sd</td><td>7.86±2.78</td><td>9.24±3.24</td><td>8.71±1.39</td><td>9.14±1.92</td><td>6.63±3.77</td><td>7.91±4.17</td><td>-0.22±0.14</td><td>-0.53±0.29</td><td>4.21±5.24</td><td>3.58±4.77</td><td><0.001</td></tr>
<tr><td colspan="13">Diaphorobacter (CLR transformation)</td></tr>
<tr><td>[-1.29 | -0.31 | 6.28 | 8.96 | 15.82]</td><td>five number summary</td><td>[6.73 | 8.23 | 8.92 | 9.59 | 10.92]</td><td>[-0.72 | 2.89 | 7.92 | 9.97 | 15.82]</td><td>[-0.58 | -0.19 | 8.16 | 8.67 | 11.7]</td><td>[-1.29 | -0.62 | -0.24 | 6.89 | 10.78]</td><td>[-0.49 | 7.09 | 8.22 | 9.6 | 11.51]</td><td>[-1.12 | 2.59 | 7.81 | 8.8 | 9.32]</td><td>[-0.61 | -0.39 | -0.16 | 0 | 0.21]</td><td>[-1.13 | -0.67 | -0.57 | -0.29 | -0.08]</td><td>[-0.99 | -0.23 | 3.59 | 9.59 | 11.99]</td><td>[-0.89 | -0.31 | -0.01 | 9.4 | 13.53]</td><td><0.001</td></tr>
<tr><td>4.54±4.86</td><td>mean±sd</td><td>8.91±1.13</td><td>6.94±5.11</td><td>5.75±4.63</td><td>2.71±4.34</td><td>7.24±3.79</td><td>5.78±4.17</td><td>-0.18±0.25</td><td>-0.54±0.32</td><td>4.63±5.14</td><td>4.27±5.3</td><td><0.001</td></tr>
<tr><td colspan="13">Gemella (CLR transformation)</td></tr>
<tr><td>[-1.37 | -0.38 | 0.11 | 9.91 | 14.47]</td><td>five number summary</td><td>[-0.82 | -0.51 | 3.67 | 9.04 | 11.07]</td><td>[-0.6 | 8.98 | 11.64 | 12.98 | 14.09]</td><td>[-0.6 | -0.5 | -0.28 | 6.34 | 11.32]</td><td>[-0.5 | 9.24 | 11.48 | 12.85 | 14.04]</td><td>[-0.59 | -0.39 | -0.23 | 2.81 | 10.29]</td><td>[-0.45 | 8.62 | 10.5 | 12.23 | 14.47]</td><td>[-0.53 | -0.16 | -0.03 | 8.42 | 9.1]</td><td>[-0.58 | 7.77 | 10.55 | 12 | 13.6]</td><td>[-0.92 | -0.57 | -0.25 | 2.97 | 8.92]</td><td>[-1.37 | -0.66 | -0.23 | 0 | 12.62]</td><td><0.001</td></tr>
<tr><td>4.75±5.54</td><td>mean±sd</td><td>4.37±5.13</td><td>10.44±3.96</td><td>2.51±4.54</td><td>10.54±3.91</td><td>1.59±3.61</td><td>9.21±4.74</td><td>2.81±4.4</td><td>8.9±4.76</td><td>1.63±3.67</td><td>1.38±4.11</td><td><0.001</td></tr>
<tr><td colspan="13">Rothia (CLR transformation)</td></tr>
<tr><td>[-1.16 | -0.29 | 7.07 | 10.33 | 15.87]</td><td>five number summary</td><td>[-0.68 | -0.39 | 7.72 | 9.22 | 10.94]</td><td>[-0.77 | 10.89 | 12.63 | 13.96 | 15.87]</td><td>[-1.12 | -0.61 | -0.18 | 8.28 | 11.32]</td><td>[-1.16 | 9.46 | 11.11 | 13.02 | 13.76]</td><td>[-0.47 | -0.3 | -0.2 | 8.05 | 10.33]</td><td>[-0.66 | 9.06 | 11.76 | 13.45 | 14.17]</td><td>[-0.63 | -0.19 | -0.03 | 6.64 | 8.8]</td><td>[-0.16 | 9.71 | 11.9 | 12.44 | 13.12]</td><td>[-0.99 | -0.5 | -0.28 | 0.04 | 10.58]</td><td>[-1.04 | -0.3 | 0.02 | 8.17 | 11.96]</td><td><0.001</td></tr>
<tr><td>5.38±5.72</td><td>mean±sd</td><td>5.69±4.72</td><td>10.86±5.56</td><td>3.35±4.92</td><td>9.57±5.14</td><td>3.47±4.74</td><td>9.73±5.09</td><td>2.38±3.85</td><td>10.23±3.84</td><td>1.22±3.7</td><td>3.26±4.9</td><td><0.001</td></tr>
<tr><td colspan="13">Staphylococcus (CLR transformation)</td></tr>
<tr><td>[-0.85 | 9.38 | 11.58 | 13.4 | 17.51]</td><td>five number summary</td><td>[8.57 | 9.7 | 11.99 | 13 | 15.06]</td><td>[10.73 | 13.16 | 14.36 | 15.85 | 16.5]</td><td>[8.87 | 9.57 | 11.34 | 12.88 | 13.93]</td><td>[7.09 | 12.48 | 14.13 | 15.9 | 17.51]</td><td>[-0.85 | 9.47 | 11.1 | 13.04 | 15.09]</td><td>[9.77 | 11.71 | 13.43 | 15.17 | 16.43]</td><td>[6.57 | 9.71 | 11.18 | 13.03 | 14.47]</td><td>[10.64 | 12.29 | 13.29 | 16.02 | 16.31]</td><td>[-0.41 | 3.72 | 9.21 | 10.3 | 12.52]</td><td>[-0.42 | 8.42 | 9.07 | 11.34 | 13.52]</td><td><0.001</td></tr>
<tr><td>11±3.96</td><td>mean±sd</td><td>11.62±2.24</td><td>14.31±1.75</td><td>11.35±1.76</td><td>13.84±2.89</td><td>10.52±4.13</td><td>13.41±2.11</td><td>11.18±2.26</td><td>13.66±2.1</td><td>7.36±4.62</td><td>8.51±3.9</td><td><0.001</td></tr>
<tr><td colspan="13">Streptococcus (CLR transformation)</td></tr>
<tr><td>[-1.2 | 8.81 | 10.85 | 15.13 | 19.2]</td><td>five number summary</td><td>[9.52 | 10.7 | 12.66 | 13.61 | 16.13]</td><td>[11.64 | 16.05 | 16.51 | 17.58 | 19.2]</td><td>[-1.2 | 9.28 | 9.93 | 11.22 | 13.5]</td><td>[11.55 | 14.82 | 16.73 | 17.23 | 17.9]</td><td>[-0.26 | 2.3 | 8.51 | 9.84 | 12.71]</td><td>[13.38 | 14.87 | 15.68 | 17.23 | 18.07]</td><td>[-0.28 | 0.1 | 7.75 | 9.25 | 11.53]</td><td>[12.9 | 14.68 | 15.62 | 16.7 | 17.58]</td><td>[-0.69 | 2.94 | 8.51 | 9.45 | 12.05]</td><td>[-0.57 | 6.13 | 9.8 | 11.28 | 14.15]</td><td><0.001</td></tr>
<tr><td>10.76±5.3</td><td>mean±sd</td><td>12.33±1.93</td><td>16.53±1.89</td><td>9.61±3.72</td><td>15.96±1.88</td><td>6.59±4.56</td><td>15.79±1.51</td><td>5.86±4.51</td><td>15.61±1.41</td><td>6.69±4.42</td><td>8.55±4.24</td><td><0.001</td></tr>
<tr><td colspan="13">Veillonella (CLR transformation)</td></tr>
<tr><td>[-1.13 | -0.26 | 7.57 | 10.56 | 15.58]</td><td>five number summary</td><td>[-0.79 | -0.39 | 8.43 | 10.49 | 13.16]</td><td>[-0.76 | 8.79 | 11.33 | 12.83 | 14.71]</td><td>[-0.65 | -0.3 | 3.62 | 9.19 | 13.76]</td><td>[-0.55 | 8.89 | 11.02 | 13.85 | 15]</td><td>[-0.62 | -0.43 | -0.21 | 7 | 9.25]</td><td>[-0.22 | 8.47 | 10.93 | 13.45 | 15.58]</td><td>[-0.68 | -0.33 | -0.14 | -0.03 | 9.15]</td><td>[-0.4 | 7.85 | 11.09 | 12.22 | 14.17]</td><td>[-0.78 | -0.26 | 6.74 | 8.62 | 11.41]</td><td>[-1.13 | -0.37 | -0.08 | 8.23 | 11.33]</td><td><0.001</td></tr>
<tr><td>5.69±5.62</td><td>mean±sd</td><td>6.48±5.49</td><td>9.71±5.17</td><td>4.68±5.37</td><td>9.84±5.28</td><td>2.4±4.09</td><td>9.72±5.14</td><td>1.27±3.55</td><td>9.68±4.33</td><td>4.6±4.75</td><td>2.39±4.49</td><td><0.001</td></tr>
</table>

Five-number summary stands for [minimum | 1st quartile | median | 3rd quartile | maximum].
To test difference in means an ANOVA test was used.
To test difference in medians a non-parametric ANOVA (Kruskal-Wallis test) was used.
All *p*-values were adjusted using Holm's method.

Supplementary Figure S2. Descriptive statistics for sequencing depth, Shannon diversity index, and centered log-ratio (CLR)–transformed relative abundances of selected genera, stratified by sample matrix, time point, and PMA treatment group. All values are rounded to two decimals.

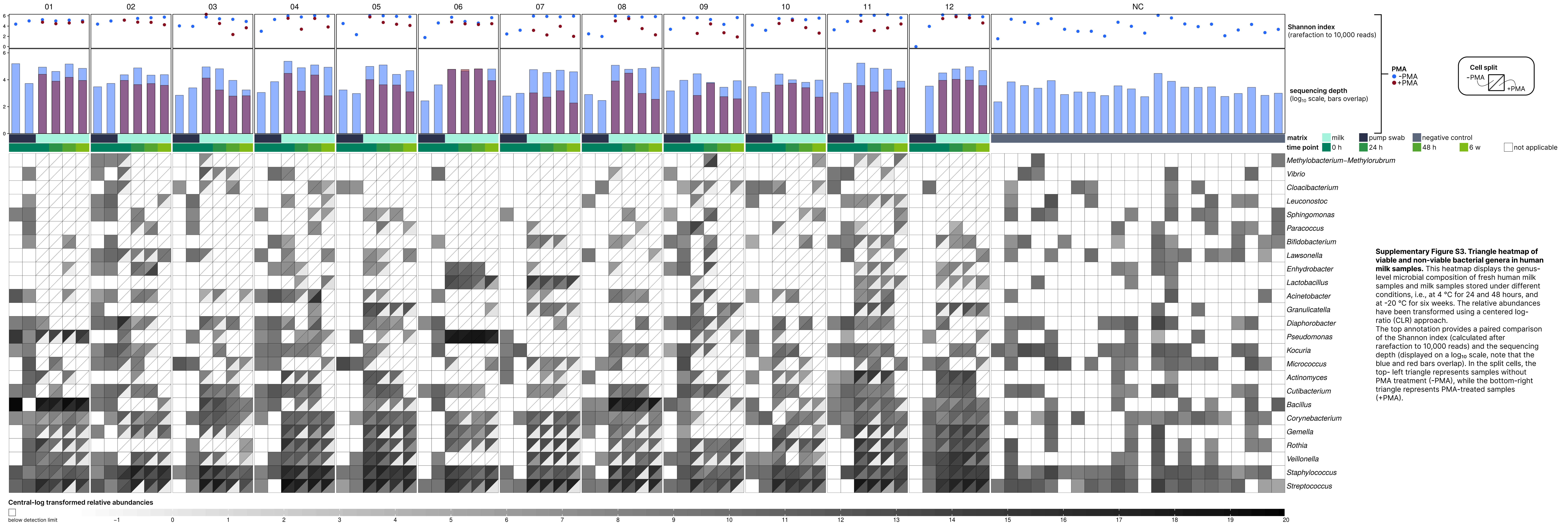

Supplementary Figure S3. Triangle heatmap of viable and non-viable bacterial genera in human milk samples. This heatmap displays the genus-level microbial composition of fresh human milk samples and milk samples stored under different conditions, i.e., at 4 °C for 24 and 48 hours, and at -20 °C for six weeks. The relative abundances have been transformed using a centered log-ratio (CLR) approach.
The top annotation provides a paired comparison of the Shannon index (calculated after rarefaction to 10,000 reads) and the sequencing depth (displayed on a log_{10} scale, note that the blue and red bars overlap). In the split cells, the top- left triangle represents samples without PMA treatment (-PMA), while the bottom-right triangle represents PMA-treated samples (+PMA).
